# Heart Disease Self-management for African American Older Adults: Outcomes of an Adapted Evidence-Based Intervention

**DOI:** 10.1093/geroni/igac053

**Published:** 2022-08-19

**Authors:** Mary R Janevic, Jessica E Ramsay, Kristi L Allgood, Aida Domazet, Shaun Cardozo, Cathleen M Connell

**Affiliations:** Department of Health Behavior and Health Education, University of Michigan School of Public Health, Ann Arbor, Michigan, USA; Department of Health Behavior and Health Education, University of Michigan School of Public Health, Ann Arbor, Michigan, USA; Department of Epidemiology and Center for Social Epidemiology and Population Health, University of Michigan School of Public Health, Ann Arbor, Michigan, USA; Detroit Area Agency on Aging, Detroit, Michigan, USA; Department of Internal Medicine, Division of Cardiology, Wayne State University School of Medicine and Detroit Medical Center, Detroit, Michigan, USA; Department of Health Behavior and Health Education, University of Michigan School of Public Health, Ann Arbor, Michigan, USA

**Keywords:** Cardiovascular disease, Disparities, Intervention, Lifestyle behaviors, PROMIS-29

## Abstract

**Background and Objectives:**

To assess the impact of an evidence-based self-management intervention adapted through a community-engaged process for African American midlife and older adults with heart disease and/or cardiovascular risk factors.

**Research Design and Methods:**

Adults 50 years and over, living in or near Detroit, MI, with diagnosed heart disease or greater or equal to two major risk factors for heart disease, were randomized to a 7-week group-format program called *Take Heart*, or a usual-care control group. *Take Heart* included education about heart disease and support for behavioral lifestyle change, using a goal-setting process based on self-regulation theory. Outcome data were collected via telephone surveys at baseline and 1 year from baseline. Primary outcomes were self-reported emergency department visits and hospitalizations in the last year. Secondary outcomes were health-related quality of life (PROMIS-29 Adult Profile) and cardiac symptom burden.

**Results:**

A total of 453 participants enrolled (74% female, 84% African American, mean age 65.4 years; 55% with diagnosed heart disease and 45% with risk factors only); 362 provided baseline and follow-up data. Using generalized linear and binomial regression models, at 12-month follow-up, there were no significant differences between intervention and control groups in ED visits or hospitalizations. Intervention versus control participants had greater improvements in PROMIS fatigue (*p* = .003) and sleep (*p* = .04) subscales as well as cardiac symptom burden (*p* = .04).

**Discussion and Implications:**

The *Take Heart* intervention was associated with modest improvements in sleep, fatigue, and cardiac symptom burden. *Take Heart* was well received and has potential for dissemination by agencies serving older adults.

Clinical Trial Registration Number: https://www.clinicaltrials.gov/ct2/show/NCT02950818

**Translational Significance:** Evidence for many behavioral interventions was derived using samples from historically centered populations. With input from community partners, we adapted an evidence-based heart disease self-management intervention for delivery by a community agency to primarily African American older adults, a group with a heavy burden of cardiovascular disease due to structural racism. The translated intervention improved health-related quality of life relative to usual-care controls, and participants consistently reported subjective benefits. Our study provides a useful template for translations of existing interventions. A toolkit of the adapted program is available for use by community agencies serving older adults.

## Background and Objectives

Cardiovascular disease (CVD) is the leading cause of death in the United States and incurs about $360B in total health care costs each year (Virani et al., 2021). Although heart disease mortality rates have been declining, the total number of heart disease deaths is increasing due to the rapid aging of the U.S. population (Sidney et al., 2019). The American Heart Association recommends self-management training for people with heart disease and/or major cardiovascular risk factors such as hypertension (Artinian et al., 2010; Barnason et al., 2017). Chronic disease self-management interventions teach skills for making lifestyle behavior changes and effectively interacting with healthcare providers, with the goal of preventing disease incidence or progression, and improving quality of life (Allegrante, 2014). Successful self-management interventions targeting health behaviors such as smoking, physical activity, diet, and weight can play a role in decreasing morbidity and mortality related to CVD (Artinian et al., 2010).

### Heart Disease Self-management and Underserved Older Adults

Compared to the general population, marginalized and minoritized populations are at higher risk for premature heart disease, have more CVD risk factors, and experience worse outcomes (Hamad et al., 2020; Mensah et al., 2005) Despite these inequities, relatively few heart disease self-management interventions have been tailored to vulnerable populations, including communities of color (Elgazzar et al., 2020). Chronic disease self-management interventions are less likely to reach people living in impoverished areas who most need them (Horrell et al., 2017) and therefore should be tailored to address common barriers, including transportation (Hardman et al., 2020).

### The *Take Heart* Intervention

Community-academic partnerships can lead to the development of interventions promoting cardiovascular health that are beneficial to communities, sustainable, and build capacity in disparity-affected communities (Elgazzar et al., 2020). Working in partnership with the Detroit Area Agency on Aging (DAAA) and the Detroit Medical Center (DMC), we adapted an evidence-based heart disease self-management intervention (*Take PRIDE*; Clark et al., 1992, Clark et al., 2000) to meet the needs of predominantly African American adults aged 50 and over in Detroit, MI. In Detroit, heart disease mortality among adults ages 50–59 is 122% higher (and 48% higher among adults aged 60–74) than similarly aged persons in the rest of Michigan (Detroit Area Agency on Aging, 2020). Heart disease is an “ambulatory care-sensitive” condition, for which hospitalizations could be prevented by interventions in primary care; yet in Detroit, 54.5% of older adults live in Medically Underserved Areas (Detroit Area Agency on Aging, 2020). These inequities are rooted in a long and persistent history of structural racism (Bailey et al., 2017), which in this predominantly African American city has produced racialized residential segregation and curtailed economic opportunities, resulting in exposure of residents to numerous health risks. These include strained social networks, decaying and/or toxic physical environments, and high levels of psychological distress (Geronimus et al., 2020).

In this study, we analyze outcomes from a randomized controlled trial (RCT) of the adapted intervention, renamed *Take Heart*, in a sample of primarily African American older adults with diagnosed heart disease or significant risk factors. The overarching purpose of *Take Heart* was to provide information and motivation to support improved lifestyle behaviors and treatment adherence, ultimately leading to decreased morbidity and improved health-related quality of life. Based on findings from trials of the original intervention, we hypothesized that relative to a usual-care control condition *Take Heart* participants would experience: (a) greater reductions in hospitalizations and emergency department (ED) use over 1 year (primary outcomes) and (b) improved cardiac symptom experience and health-related quality of life, as measured by the PROMIS Adult Profile subscales (secondary outcomes). We also report data on participant satisfaction and perceptions of intervention benefits.

## Research Design and Methods

*Take Heart* was approved by the University of Michigan Institutional Review Board (HUM00092196) and registered in ClinicalTrials.gov (NCT02950818).

### Intervention Adaptation and Content

Starting with the original curriculum developed by Clark et al. (2009), we used the Replicating Effective Programs framework (Kilbourne et al., 2007) to integrate input from diverse stakeholders into the new design, so that it would meet the needs and preferences of older Detroiters (see Ramsay et al., 2019 for details of the adaptation process). Based on feedback from our priority population, we adapted the program in several ways. For example, we added more content about stress management, a list of local resources for older adults, and, in response to a reported dearth of information from providers, we added simple explanations of the pathophysiology of common heart conditions. To accommodate aging-related challenges, we used large-font materials and provided transportation. Content and examples used in the education sessions—for example, exercise/activities and nutrition tips—were also tailored to be appropriate for older adults.

Because *prevention* of heart disease, in addition to management of existing disease, was a high priority for our community partners, we expanded initial eligibility criteria to include individuals who did not yet have a diagnosed heart condition but who had significant risk factors. We also included adults as young as 50 (compared with 60 in the original trials), given accelerated aging in African American adults due to the health-damaging effects of structural racism (Bailey et al., 2017; Levine & Crimmins, 2014). This process of accelerated aging (as demonstrated in the shorter telomere length in Black vs. White women in midlife; Geronimus et al. 2010) is also seen in the early onset and accumulation of chronic health conditions among African Americans, including cardiovascular conditions (Quiñones et al., 2019), and the strikingly higher cardiovascular mortality rates in African Americans during midlife (Mensah & Brown 2007). African Americans are also far more likely to also have cardiovascular risk factors at younger ages (Graham, 2015). These are also more severe; for example, African Americans ages 45–64 are more than five times as likely as their White counterparts to be hospitalized for hypertension.

*Take Heart* was delivered primarily at the DAAA; two series (i.e., a set of seven weekly classes and two 1:1 phone calls) took place at senior housing buildings. Classes were led by a trained community health educator employed by the DAAA. Topics included understanding heart medications and taking them correctly, and lifestyle behaviors including smoking, stress management, nutrition, and physical activity. Two innovative features were an “Ask a Cardiologist” segment where study team member (S. Cardozo) was available by telephone for a portion of one session to answer participants’ questions about heart disease, and an interactive cooking demonstration led by a nutrition-focused community agency. The primary behavioral change mechanism of *Take Heart* was a goal-setting process based on self-regulation theory (Clark et al., 2001), and participants selected one or more self-management areas (e.g., healthy eating, exercise) for setting behavior change goals. *Take Heart* content included core theory-based components of the original intervention and of cardiac rehabilitation developed by Kabboul et al. (2018) as well as the AHA’s Life Simple 7 prevention guidelines (Virani et al., 2021).

Participants were recruited between December 10, 2016 and October 18, 2019. Recruitment strategies were *community-based* (information sessions, flyer distribution, DAAA referrals, and word of mouth); *clinic-based* (tabling at DMC’s Rosa Parks Geriatric Center and Wayne State University’s Cardiology Clinic); and through *electronic medical records* (potentially eligible patients from these clinics were identified by S. Cardozo and contacted by study staff to assess eligibility and interest). Additional information on recruitment is available (Ramsay et al., 2020).

#### Eligibility criteria

At least 50 years of age, self-report of one or more cardiac diagnoses (atrial fibrillation, myocardial infarction, valvular disease, pulmonary hypertension, angina, congestive heart failure, or peripheral vascular disease) *or* at least two major cardiovascular risk factors (high cholesterol, high blood pressure, diabetes, Stage 3 or 4 chronic kidney disease, and smoking), and ability to participate in a group in-person (transportation was provided if needed) and individual telephone education sessions. After baseline data collection, participants were randomized in a 1:1 ratio to intervention or usual-care control groups using random block sizes of 4 or 6.

All outcomes were collected via telephone interviews conducted by trained staff at baseline and 12 months from baseline. Interviews lasted about one hour and consisted of standardized measures of health, functioning, and demographics. Participants were offered $20 for each interview. Study data were collected and managed using REDCap (Harris et al., 2019). To maximize retention, participants were contacted by telephone up to 10 times each. At that point, an “unable to contact” letter was mailed inviting participants to contact study staff; participants not responding to the letter were marked as lost to follow-up.

Intervention group participants who missed the first session of the series were offered enrollment in two alternative series. Participants who subsequently missed both of these were withdrawn from the study. In a few cases, intervention participants who withdrew but expressed an interest in learning intervention content were given the option of attending the control group half-day workshop.

### Primary Outcomes: Health Care Utilization

In baseline and follow-up telephone surveys, participants were asked: “During the past 12 months, how many times have you gone to a hospital emergency room about your own health (this includes emergency room visits that resulted in a hospital admission and those that did not)?” and “During the past 12 months, how many times did you stay overnight in a hospital for something related to your own health?” Self-report of medical utilization is a “viable and cost-effective” method among older adults according to Lubeck & Hubert (2005), who obtained a weighted kappa statistic of 1.0 (indicating near-perfect agreement) for concordance of self-reported hospital and ED visits with medical records over one year in older adults (median age 77 years). In a study among older adults with previous myocardial infarction, self-reported hospitalizations verified with administrative records demonstrated high sensitivity, specificity, and accuracy for up to 12 months (Seidl et al., 2016).

### Secondary Outcomes: Health-Related Quality of Life

The *PROMIS-29 Adult Profile* (Cella et al., 2019) measures eight domains of HRQoL with four items each on Likert scales: physical functioning, anxiety, depression, fatigue, sleep disturbance, ability to participate in social roles/activities, pain interference, and pain intensity (0 = no pain at all to 10 = worst imaginable pain). Raw subscale scores were summed and converted to *T*-scores (a standardized score with a mean of 50 and *SD* of 10) using the conversion tables provided at HealthMeasures.net. PROMIS measures have established high reliability and construct validity (Cella et al., 2019).

The *cardiac symptom subscale* of the Symptom and Health Problem Profile (Janz et al., 1999) asks about the frequency of chest pain/discomfort; shortness of breath; waking up from sleep because of chest pain or pressure; waking up from sleep because of shortness of breath or difficulty breathing; and irregular heartbeat or palpitations (not present, once or twice/week, a few times/week, once/day, several times/day) in the prior 12 months. The test–retest reliability of this instrument is .79 (Clark et al., 1997). Symptom frequency (0–4) was summed, yielding an overall symptom burden score that ranged from 0 to 20.

#### Additional variables

Participants indicated yes/no to being diagnosed with each of seven common chronic conditions; self-rated health (excellent to poor); sex; age; racialized group and ethnicity; marital status; employment status; annual income; and educational attainment.

#### Participant satisfaction

Responses to the following items for intervention group participants were on Likert scales: “Attending the *Take Heart* education sessions increased my understanding of heart health.” “The *Take Heart* program motivated me to begin or continue making healthy lifestyle changes.” “After completing the *Take Heart* program, how motivated do you feel to continue making healthy lifestyle changes or maintain changes that you have already made?” “Would you recommend the *Take Heart* program to others?” We also collected open-ended responses to the following items: “What did you like best/least about the *Take Heart* program?” “What else would you like to share with us about your experience in *Take Heart*?”

### Statistical Analysis

Analyses were conducted using SAS, version 9.4 (SAS Inc., Cary, NC). Univariate analysis was used to determine the functional form of each variable and to calculate sample descriptive statistics at baseline. Independent-sample *t*-tests were used to determine baseline differences between arms in means of continuous variables, and chi-square tests for categorical variables.

Statistical model forms were selected based on the distribution of the outcomes of interest, and each model was analyzed as a complete-case analysis. As such, each model includes a slightly different sample size due to missing data. All models controlled for sex because of a statistically greater percentage of men in the intervention versus control group (32% vs. 20%; see Table 1). We also controlled for educational attainment and age because of the known associations between these variables and the outcomes.

**Table 1.** Descriptive Statistics of *Take Heart* Participants, Overall and by Group Assignment

Respondent Characteristics		Overall *N* = 453^a^	Intervention *n* = 228	Control *n* = 225	*p*-Value for between-group comparisons^b^
Age in years, mean (*SD*)		65.4 (±9.0)	65.5 (±9.3)	65.3 (±8.6)	.8040
Sex, *n* (%)	Male	118 (26.0)	73 (32.0)	45 (20.1)	.0096
	Female	334 (73.7)	155 (68.0)	179 (79.9)	
Race, *n* (%)	Hispanic	4 (0.9)	2 (0.9)	2 (0.9)	.9626
	Non-Hispanic Black	380 (83.9)	193 (85.8)	187 (85.0)	
	Non-Hispanic White	20 (4.4)	10 (4.4)	10 (4.5)	
	Other^c^	41 (9.1)	20 (8.9)	21 (9.5)	
Education, *n* (%)	<High school	53 (11.7)	23 (10.2)	30 (13.3)	.1794
	High school grad or GED	111 (24.5)	55 (24.3)	56 (24.9)	
	Some college	194 (42.8)	107 (47.3)	87 (38.7)	
	College graduate or higher	93 (20.5)	41 (18.1)	52 (23.1)	
Marital status, *n* (%)	Married/partnered	57 (12.6)	24 (10.8)	33 (14.7)	.2774
Employment status, *n* (%)	Employed	45 (9.9)	26 (11.6)	19 (8.4)	.5600
	Retired	226 (49.9)	107 (47.8)	119 (52.7)	
	Unemployed	86 (19.0)	45 (20.1)	41 (18.1)	
	Other	95 (21.0)	46 (20.5)	49 (21.7)	
Self-rated health, *n* (%)	Excellent	8 (1.8)	2 (0.9)	6 (2.7)	.3644
	Very good	41 (9.1)	22 (9.7)	19 (8.4)	
	Good	146 (32.2)	79 (34.8)	67 (29.8)	
	Fair	177 (39.1)	89 (39.2)	88 (39.1)	
	Poor	80 (17.7)	35 (15.4)	45 (20.0)	
Income	≤$15,000	250 (58.9)	134 (62.3)	116 (55.2)	.0686
	$15,001–$40,000	134 (31.5)	67 (31.2)	67 (31.9)	
	>$40,000	41 (9.6)	14 (6.5)	27 (12.9)	
Heart disease diagnoses, *n* (%)	Atrial fibrillation	86 (19.0)	42 (18.4)	44 (19.6)	.7582
	Myocardial infarction	79 (17.4)	41 (18.0)	38 (16.9)	.7591
	Valvular disease	34 (7.5)	12 (5.3)	22 (9.8)	.0682
	Pulmonary hypertension	18 (4.0)	11 (4.8)	7 (3.1)	.3506
	Angina	77 (17.0)	33 (14.5)	44 (19.6)	.1499
	Congestive heart failure	78 (17.2)	33 (14.5)	45 (20.0)	.1193
	Peripheral vascular disease	53 (11.7)	27 (11.8)	26 (11.6)	.9244
	Other heart disease condition	15 (3.3)	9 (3.9)	6 (2.7)	.4463
Risk factors, *n* (%)	High cholesterol	354 (78.1)	184 (80.7)	170 (75.6)	.1851
	High blood pressure	414 (91.4)	210 (92.1)	204 (90.7)	.5852
	Diabetes	197 (43.5)	109 (47.8)	88 (39.1)	.0619
	Chronic kidney disease	26 (5.7)	10 (4.4)	16 (7.1)	.2125
	Current smoker	133 (29.4)	63 (27.6)	70 (31.8)	.2125
Comorbidities, *n* (%)	Depression	171 (37.7)	91 (40.1)	80 (35.7)	.6324
	Cancer	70 (15.5)	35 (15.5)	35 (15.6)	.8504
	Prediabetes	188 (41.5)	104 (45.8)	84 (37.8)	.1374
	Stroke	75 (16.6)	33 (14.6)	42 (18.8)	.4240
	Arthritis	304 (67.1)	146 (64.0)	158 (71.5)	.0311
	COPD	121 (26.7)	50 (22.3)	71 (32.0)	.0674
	Asthma	121 (26.7)	57 (25.3)	64 (29.0)	.6375
Sum of comorbidities, mean sum (*SD*)		2.3 (1.4)	2.3 (1.4)	2.4 (1.4)	.2825

*Notes:* GED = general educational development; COPD = chronic obstructive pulmonary disease.

^a^Not all counts add to 453 due to missing values.

^b^Differences in means of continuous variables were determined using *t*-tests; difference in percentages with chi-square tests.

^c^“Other” includes multiracial and American/Alaskan Native. All but one participant in this category marked a racial group as African American/Black.

For count variables (emergency department visits and hospital admissions), many cases had zero values, right-skewing these outcomes. We examined each model using zero-inflated and noninflated Poisson and negative binomial regression analyses. After examining goodness of fit values using the Voung and Clarke statistics, a negative binomial regression analysis was selected. The PROMIS-29 subscales and cardiac symptom frequency were analyzed using generalized linear regression models controlling for age, sex, and educational attainment. Each continuous outcome had a non-normal distribution; however, the error terms were normally distributed and all remaining assumptions for linear regression were met. As a last step, we repeated the primary analyses stratified by sex and heart disease status to facilitate comparisons to findings from the original *Take PRIDE* studies from which *Take Heart* was adapted.

For participant satisfaction close-ended items, we report frequencies. Open-ended responses were pooled across items due to overlapping content and then coded and categorized into themes.

## Results

A total of 453 participants were randomized into intervention (*n* = 228) or control groups (*n* = 225). As shown in Table 1, enrolled participants identified as primarily African American (84%) and female (74%). Most participants were between the ages of 50 and 69 years (70%), were not married or partnered (87%), were retired (50%). Over 60% of the sample completed at least some college, yet 59% reported an annual household income of $15,000 or less (59%). Just over half of the sample (*n* = 247; 54.5%) had a heart disease diagnosis; the remaining participants had cardiovascular risk factors only. At baseline, there were more women in the control versus intervention group (68% vs. 80%; *p* = .01). There were no between-group differences for any specific heart disease diagnosis or risk factor. There was a higher prevalence of arthritis in the control group versus intervention group (72% vs. 64%; *p* = .03).

**Figure 1.**
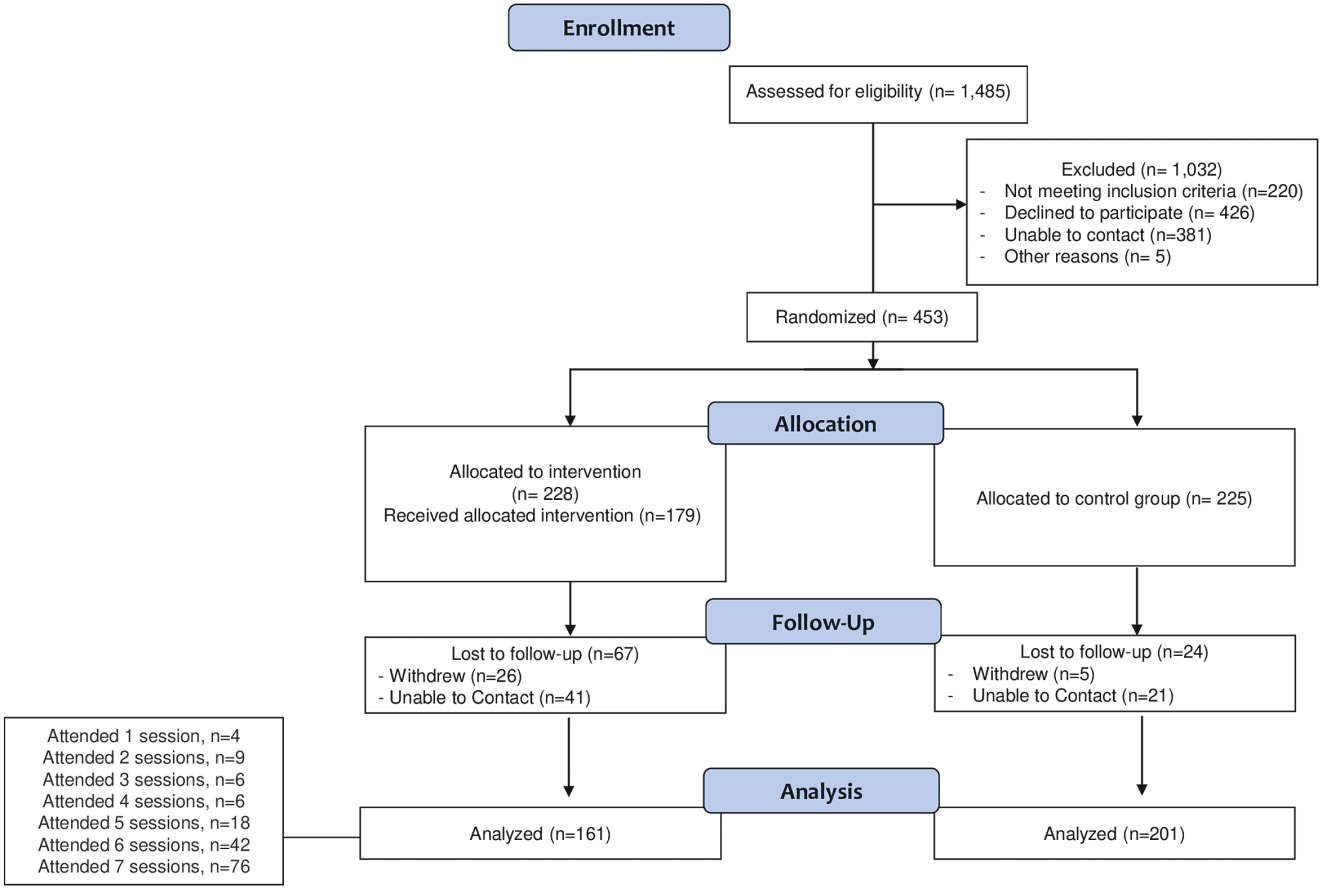
CONSORT flow diagram of participants.

A total of 67 participants (29%) withdrew (*n* = 26) or were lost to follow-up (*n* = 41) in the intervention group and 24 participants (11%) withdrew (*n* = 5) or were lost to follow-up (*n* = 21) in the control group (Figure 1). Among the 67 intervention group members without follow-up data, 18 attended at least one intervention session. The most common reasons given for withdrawal were lack of time and “no longer interested.” Compared with completers, noncompleters were more likely to be male, have less than a high school education, and have a history of myocardial infarction and pulmonary hypertension (Supplementary Table 3).

Table 2 displays the adjusted means, adjusted ordinal odds ratios, and between-group differences in means at 1-year follow-up. For all but one outcome, the intervention group reported lower healthcare utilization, lower (i.e., better) PROMIS scores, and lower cardiac symptom burden at 1-year follow-up; a few of these outcomes reached statistical significance. There were no differences in *emergency room visit*s or *hospital admissions* at 12-month follow-up. As a sensitivity analysis, ordered categories of these outcomes were analyzed with an ordinal logistic regression analysis. The score test (Hosmer & Lemeshow, 2000) was used to examine the proportional odds assumption. For emergency department admissions, the proportional odds assumption was not met; therefore, multinomial regression was used. Results of the ordinal analysis of both outcomes were consistent with the negative binomial regression.

**Table 2.** Adjusted Odds Ratios, Means, and Mean Differences in Primary Outcomes at 12 Months by Group Assignment, *n* = 362

Respondent characteristics	Intervention (*n* = 161)	Control (*n* = 201)	
	Adjusted^a^ Mean (95% CI)	Adjusted^a^ Mean (95% CI)	Odds Ratio^b^/Difference^c^^-^^e^ (95% CI), *p*-value
Health care utilization			
Emergency department visits (count)^c^	0.98 (0.98, 1.60)	1.48 (1.19, 1.84)	0.85 (0.64, 1.14), *p* = .1483
Hospital admissions (count)^c^	2.55 (1.64, 3.97)	2.74 (1.76, 4.27)	0.93 (0.54, 1.60), *p* = .8014
PROMIS-29^d^			
Physical functioning	33.0 (31.9, 34.2)	34.1 (33.0, 35.2)	−1.07 (−2.45, 0.30), *p* = .1243
Anxiety	54.1 (52.5, 55.7)	54.1 (52.6, 55.6)	0.002 (−1.91, 1.91), *p* = .9987
Depression	51.0 (49.4, 52.6)	51.3 (49.8, 52.7)	−0.26 (−2.12, 1.60), *p* = .7848
Fatigue	51.3 (49.8, 52.9)	54.2 (52.7, 55.6)	−2.822 (−4.70, −0.95), *p* = .0033
Sleep	51.9 (51.3, 52.5)	52.6 (52.1, 53.2)	−0.727 (−1.43, −0.02), *p* = .0429
Social	38.9 (34.4, 40.5)	40.1 (28.6, 41.5)	−1.129 (−2.98, 0.07), *p* = 0.2300
Pain interference	57.6 (55.8, 59.4)	58.9 (57.2, 60.6)	−1.333 (−3.47, 0.80), *p* = 0.2202
Pain intensity	5.1 (4.6, 5.5)	5.4 (4.9, 5.8)	−0.318 (−0.85, −0.22), *p* = .2435
Cardiac symptom burden^d^	1.96 (1.35, 2.58)	2.74 (2.17, 3.32)	−0.780 (−1.51, −0.05), *p* = .0356

*Notes:* CI = confidence interval.

^a^All models are adjusted for age, sex, and education.

^b^Adjusted odds ratios for ordinal outcomes are estimated using ordinal logistic regression.

^c^Adjusted means for count outcomes are estimated using negative binomial regression.

^d^Adjusted means for continuous outcomes are estimated using generalized linear regression.

^e^Higher values of all outcomes indicates poorer health. All differences are calculated as Intervention mean − Control mean, negative scores indicate lower scores among the Intervention group.

At follow-up, the PROMIS-29 *fatigue subscale score* was significantly lower in the intervention group compared with the control group (between-group difference: −2.8 [−4.70, −0.95], *p* = .00), as was the *sleep subscale score* (between-group difference: −0.73 [−1.43, −0.02], *p* = .04). We also observed a significantly lower *cardiac symptom burden* score in the intervention group (between-group difference: −0.780 [−1.506, −0.054], *p* = .04).

### Stratified Analyses

For cardiac symptom burden, the between-group difference was marginally significant (*p* = .0551) for those with cardiac risk factors but not for those with diagnosed heart disease, and among men (*p* = .0472) but not women. For the PROMIS sleep subscale, the between-group difference was marginally significant (*p* = .0576) for those with diagnosed heart disease but not with risk factors only, or within men or women. For the fatigue subscale, the intervention group had a significantly better score (*p* = .0031) among women but not men, as did intervention participants with risk factors (*p* = .0252) but not those with heart disease (Supplementary Tables 1 and 2).

### Participant Satisfaction

Among the 161 intervention group participants, 97% strongly agreed or agreed that *Take Heart* motivated them to begin or continue making healthy lifestyle changes; 60% said they were “extremely” or “very” motivated to continue making or maintaining these changes; and 99% strongly agreed or agreed that *Take Heart* increased their understanding of heart health. One hundred percent of participants would recommend the program to someone else. Analysis of responses across open-ended questions revealed similarly positive views about the program, with the following themes most strongly endorsed (Table 3): Participants valued knowledge and information acquired (96 comments); benefited socially from the group dynamics (62 comments); learned from peers (61 comments); felt empowered/motivated to make changes (60 comments); felt very satisfied with overall program (60 comments); and felt that the instructor was excellent (60 comments). The most common critical responses (not shown in table) were that the program was “too short” (sessions and/or series length; 41 comments) and some participants who used the transportation service experienced difficulties like long waits (14 comments).

**Table 3.** Common Themes From Open-Ended Program Satisfaction Questions (*n* = 161)

Theme	Illustrative responses
Valued knowledge and information acquired	“The information that they gave. The information they gave was good information. It kept me motivated because I’m going to the gym today.” “She liked learning that even though she hasn’t had a heart attack that just having risk factors (e.g., diabetes or high blood pressure) that those things can lead to a heart attack. She didn’t know that before the class.”
Benefited from the group format	“Well I hope all enjoyed the class as much as I did, plus I enjoyed being with the senior because you have to remember senior don’t get out like they used to when they were younger so it is really just a joy for us to get out and be among people that we can relate to.” “The whole group setting. Just being able to speak to other people in the same situation being there with the same intentions to improve their life.”
Empowered/motivated to make changes	“I loved the way they motivated me to set my own goals and get me motivated to do things I needed to do in my life.” “It has given me the power to reset what I do to live healthier. I am hyped, I am geeked, I am elated from the information. I was empowered by it. It has motivated me and has kept me motivated. It was just phenomenal.”
Very satisfied with overall program/nothing participant didn’t like	“[I liked] the information that was given, the health educator, just everything about it. The goal setting, the freebies, the whole program. The health educator’s enthusiasm, consistency, friendliness, and knowledge about topics. Lifesaving, life changing.” “If I could do it all over again, I would. I really would.”
Excellent instructor	“Our instructor was GREAT—she made the class feel so close, like we were just friends, felt comfortable speaking in front of one another, we interact together very good—you felt like if you asked a question, you weren’t asking something out of place.” “She took time to explain, she didn’t just give you the paperwork she actually explained to us.”

## Discussion and Implications

Working in conjunction with community partners, we adapted an evidence-based heart disease self-management intervention to meet the needs of older adults living in a primarily African American, economically deprived community with a heavy burden of CVD. A randomized controlled trial of the “*Take Heart*” program did not demonstrate an effect on self-reported emergency department visits or hospitalizations at 1-year follow-up. The intervention also targeted health-related quality of life (HRQoL) and was associated with reduced fatigue and improved sleep, and decreased cardiac symptom burden.

These findings are in alignment with a recent Cochrane systematic review that examined education-based interventions for heart disease across 22 trials (Anderson et al., 2017). The review did not find an impact of educational interventions on hospitalizations, subsequent myocardial infarction, or all-cause mortality, but did find some evidence of an impact on HRQoL, although there was not a consistent pattern across studies in terms of which domains of HRQoL improved. The authors concluded that educational interventions alone might not be sufficient to impact key health outcomes among individuals with heart disease and recommended that education be implemented as part of a multicomponent program that also includes exercise and psychological support.

Sleep and fatigue are important patient-centered outcomes and *Take Heart* participants improved in both areas relative to randomized controls. Although sleep hygiene was not a specific focus of the intervention, it is possible that the program’s emphasis on becoming more active and reducing stress resulted in better sleep and less fatigue. In addition, dietary changes prompted by the intervention, resulting in improved nutrition, may have contributed to reduced fatigue (Azzolino et al., 2020) At follow-up, the adjusted fatigue subscale *T*-score mean of the control group was nearly 3 points higher (indicating more fatigue) than the intervention group, a clinically meaningful difference (Beaumont et al., 2021). Fatigue is closely linked to daily functioning and is a common symptom in stable coronary heart disease (Eckhardt et al., 2014). It is also a predictor of adverse health events including falls and functional decline (Azzolino et al., 2020). Given that fatigue is almost twice as common in older adults of color relative to Whites (Meng et al., 2010), the decrease in fatigue reported by *Take Heart* participants is particularly relevant. Stratified analysis showed that the intervention effect on fatigue was stronger in the group of participants who did not have diagnosed heart disease, suggesting that different intervention strategies might be needed to reduce fatigue among individuals with greater cardiac morbidity.

As noted earlier, *Take Heart* was adapted from the “*Take PRIDE*” (Clark et al., 1992) and “*Women Take PRIDE*” interventions (Clark et al., 2000), to be suitable for delivery in a community-based setting by agency staff. As noted above, we made changes to intervention content and format, as well as to study eligibility criteria, in response to input from community partners and members of the priority population. Specifically, the original studies included only participants with diagnosed heart disease who were primarily White and recruited from medical practices. In *Take Heart*, we also included individuals who had cardiovascular risk factors, and we recruited primarily from community settings in an underserved community of color. Despite fairly high educational attainment in our sample as a whole, many participants had very low income, with 59% having an annual income below $15,000, illustrating the phenomena of diminished gains from education reaped by Black versus White Americans (Assari, 2018).

Notably, the adapted program retained many of the original elements, including the group format; use of a video depicting a “model” participant to build self-efficacy for behavior change; core theory-based components including the PRIDE process and goal-setting; and a focus on the self-management areas of physical activity, nutrition, stress, and medication management. Given the ongoing need to translate evidence-based interventions to new populations and settings, it is instructive to compare the results of the adaptation to the original. In the “Women *Take PRIDE*” trial, medical record data showed that participants experienced 46% fewer heart-related hospital admissions than controls (Wheeler et al., 2003). In the current study, we did not observe an effect on self-reported, all-cause hospital admissions or ED use. It is possible that this outcome may take more time to develop, as intervention-related behavior changes gradually exert their protective effect on health. Notably, the time period examined in Wheeler et al.’s (2003) study was 2 years, compared with 1 year in the current study. In stratified analyses, we examined outcomes within the subgroup of participants with a heart disease diagnosis (like the Women *Take PRIDE* sample) versus those with risk factors only. Results were similar in both subgroups to that of the full sample, suggesting that this particular finding was not replicated (Supplementary Table 1).

Findings regarding HRQoL also differed between the original and current studies. The original *Take PRIDE* intervention (Clark et al., 1992) had a positive impact on the psychosocial dimension of the Sickness Impact Profile (Bergner et al., 1981) at 1-year follow-up, but not on sleep or fatigue, as was found in the current study, using the PROMIS-29. Women *Take PRIDE* was associated with decreased cardiac symptom burden at 1 year but failed to affect the psychosocial or physical subscales of the Sickness Impact Profile. When we analyzed subgroups of *Take Heart* participants with diagnosed heart disease versus risk factors only, we found that the risk-factor-only group showed a marginally significant reduction in cardiac symptom frequency, whereas those with diagnosed heart disease did not, suggesting that symptoms are less amenable to change in the group with more advanced illness (Supplementary Table 1).

Despite mixed results for our primary and secondary outcomes, satisfaction items showed that the overwhelming majority of participants had a strongly positive experience with *Take Heart*, and reported many benefits. These included being more knowledgeable and educated about heart disease, being able to set and achieve behavioral goals, and having gained from the camaraderie and wisdom of the participants in their groups. Indeed, based on the satisfaction data collected for this study, *Take Heart* appears to be a potent intervention for increasing knowledge and awareness about heart disease and self-management and bringing about empowerment and lifestyle change in this vulnerable population.

It is possible, then, that our standardized survey measures simply did not capture real effects of the program. It is also possible that its impact would best be captured over a longer-term period than we were able to measure, as participants apply new self-management skills to emerging health challenges, and as newly adopted health behaviors become ingrained habits. Regardless, participant feedback made clear that there was much about the content and format of *Take Heart* that was both appealing and useful to participants, including the program features of the cardiologist “phone-in” and the cooking demonstration. Moreover, as we discuss elsewhere (Ramsay et al. 2019), study recruitment was completed ahead of schedule due to strong interest among potential participants in receiving education about heart disease. By these metrics, *Take Heart* was highly successful and can be used as a model for future interventions with this priority population.

### Limitations

Notable study limitations are as follows. First, we did not measure outcomes immediately following the intervention, when its effects may have been strongest. We selected a single 1-year follow-up point to minimize participant data collection burden and optimize the window to see health care utilization outcomes accrue. Second, a significant shortcoming is that our health care utilization (HCU) outcomes were based on self-report rather than electronic medical records. Although major events such as ED and hospital visits are generally recalled accurately in older, chronically ill populations, there may be some overreporting bias (Ritter et al., 2001). This bias, however, would be equally present in intervention and control groups. In future studies, assessing compliance with outpatient clinic appointments may be warranted, given that these visits are vital to cardiovascular care but, anecdotally, are frequently missed.

Second, 20.1% of our participants withdrew or were lost to follow-up, compared with an average of 11.4% in the Anderson et al.’s (2017) review of trials of patient education for heart disease. Moreover, we observed differential attrition by group assignment (10.6% in the control group vs. 29.4% in the intervention group). Intervention group participants may have been more likely to drop out due to the burden of class attendance (e.g., transportation problems, caregiving responsibilities). This may have rendered the two treatment conditions less comparable, making observed findings harder to interpret. The participants who dropped out were less healthy than those who remained, which may have made them either more or less likely to benefit from the intervention. Finally, we did not collect physiological data about potential reduction of risk factors, such as blood pressure or cholesterol.

Several strengths are worth noting. First, we were able to recruit a large number of participants from a vulnerable group that is underrepresented in RCTs of behavioral interventions. Second, we were able to engage community partners in the adaptation and implementation of a successful heart disease self-management program that can be further disseminated by local agencies. Finally, women are much more likely than men to participate in health promotion and disease management interventions (Smith et al., 2018), yet we were able to enroll a substantial proportion of men (26% of total sample). African American men are burdened by a shorter life expectancy than White men; about 32% of this difference is due to CVD (Carnethon et al., 2017). The added vulnerability of Black men to poor health outcomes makes the test of *Take Heart* especially valuable in this group. Male, but not female, participants showed a significant reduction in cardiac symptoms (Supplementary Table 2).

## Conclusions/Future Directions

Ideally, an RCT of an intervention provides evidence that program participation results in improved outcomes. In the case of *Take Heart*, our evidence is mixed. Although participants reported less fatigue, improved sleep, and reduction in cardiac symptoms compared with controls, we found no evidence on impact on self-reported health care utilization. Yet the benefits of disseminating a well-received program like *Take Heart* to individuals who are eager to participate are likely sufficient to merit the program’s inclusion in a menu of options for implementation in practice. The fact that participants with significant cardiac risk factors but who had not yet been diagnosed with heart disease benefitted the most from the program suggests that this early-stage group might be an especially appropriate audience for *Take Heart*. We have developed a toolkit which will be available to community-based organizations and will include the curriculum/materials from each class as well as guidance for facilitators. Given the aging of our population, increasing prevalence of cardiac morbidity related to COVID-19 infection, and the persistent and deeply rooted inequities in morbidity and mortality, increasing access to successful programs adapted for underserved groups of older adults seems timely and appropriate.

## Supplementary Material

igac053_suppl_Supplementary_Material
