# Supplementary material for: Heart Disease Self-management for African American Older Adults: Outcomes of an Adapted Evidence-Based Intervention
Source: Innov Aging. 2022 Aug 19;6(7):igac053. doi: 10.1093/geroni/igac053 (PMC9579718; doi:10.1093/geroni/igac053)
Supplement: igac053_suppl_Supplementary_Material [file igac053_suppl_supplementary_material.docx]

**Supplementary Table 1.**

*Adjusted Means and Mean Differences in Primary Outcomes by Group Assignment & Cardiovascular Disease (CVD)/Risk Status, n=362*

| **Respondent Characteristics** | **Among those with a Diagnosed CVD Condition, *n*=199** | | | **Among those with No CVD conditions, but Reported CVD Risk Factors, *n*=161** | | |
| --- | --- | --- | --- | --- | --- | --- |
|  | **Intervention** | **Control** |  | **Intervention** | **Control** |  |
|  | ***n* = 84** | ***n* = 115** |  | ***n* = 76** | ***n* = 85** |  |
|  | **Adjusted^a^ Mean**  **(95% CI)** | **Adjusted^a^ Mean**  **(95% CI)** | **Difference^c-e^ (95% CI),**  ***p*-value** | **Adjusted^a^ Mean**  **(95% CI)** | **Adjusted^a^ Mean**  **(95% CI)** | **Difference^c-e^ (95% CI), *p*-value** |
| **Healthcare Utilization** |  |  |  |  |  |  |
| Emergency Department Visits (count)^c^ | 1.45  (1.07, 1.96) | 1.76  (1.34, 2.31) | -0.195 (-0.569, 0.179), p = 0.3061 | 1.04  (0.71, 1.54) | 0.98  (0.68, 1.41) | 0.065 (-0.392, 0.521), p = 0.7811 |
| Hospital Admissions (count)^c^ | 3.36  (1.97, 5.72) | 3.27  (1.95, 5.49) | 0.028 (-0.642, 0.697), p = 0.9357 | 1.39  (0.70, 2.74) | 1.86  (0.93, 4.17) | -0.295 (-1.189, 0.600), p = 0.5182 |
| **PROMIS-29^d^** |  |  |  |  |  |  |
| Physical Functioning | 33.9  (32.3, 35.4) | 34.6  (33.2, 36.0) | -0.708 (-2.607, 1.190), p = 0.4642 | 32.2  (30.4, 34.0) | 33.4  (31.7, 35.1) | -1.179 (-3.223, 0.866), p = 0.2565 |
| Anxiety | 54.0  (51.7, 56.3) | 53.7  (51.6, 55.9) | 0.251 (-2.530, 3.031), p = 0.8591 | 54.7  (52.4, 57.1) | 57.8  (52.6, 56.9) | -0.040 (-2.679, 2.600), p = 0.9763 |
| Depression | 51.7  (49.5, 53.8) | 50.7  (48.7, 52.8) | 0.943 (-1.686, 3.572), p = 0.4803 | 50.5  (48.1, 52.9) | 51.9  (49.7, 54.1) | -1.462 (-4.172, 1.248), p = 0.2881 |
| Fatigue | 52.7  (50.5, 54.9) | 55.0  (-5.0, 0.37) | -2.305 (-4.982, 0.372), p = 0.0911 | 50.1  (47.7, 52.4) | 53.1  (50.9, 55.2) | -3.000 (-5.622, -0.378), p = 0.0252 |
| Sleep | 51.9  (51.1, 52.7) | 52.8  (52.1, 53.6) | -0.946 (-1.922, 0.031), p=0.0576 | 51.9  (51.0, 52.9) | 52.4  (51.6, 53.3) | -0.488 (-1.563, 0.587), p = 0.3708 |
| Social | 39.7  (37.6, 41.8) | 41.0  (39.0, 43.0) | -1.239 (-3.835, 1.358), p=0.3478 | 38.2  (35.8, 40.6) | 38.7  (36.4, 40.9) | -0.449 (-3.140, 2.241), p = 0.7418 |
| Pain Interference | 58.8  (56.4, 61.2) | 59.5  (57.3, 61.8) | -0.743 (-3.694, 2.208), p=0.6200 | 56.6  (53.8, 60.4) | 57.7  (55.1, 60.4) | -1.140 (-4.315, 2.035), p = 0.4791 |
| Pain Intensity | 5.45  (4.84, 6.06) | 5.51  (4.93, 6.09) | -0.061 (-0. 820, 0.699), p = 0.8750 | 5.63  (3.94, 5.32) | 5.10  (4.46, 5.74) | -0.465 (-1.247, 0.316), p = 0.2411 |
| Cardiac symptom burden^d^ | 2.61  (1.67, 3.55) | 3.18  (2.29, 4.07) | -0.568 (-1.724, 0.587), p = 0.3330 | 1.27  (0.60, 1.95) | 2.02  (1.40, 2.64) | '-0.748 (-1.512, 0.017), p = 0.0551 |

*n=2 are missing risk factor data, both are coded as not having CVD, 1 in each group (intervention & control)

^a^ All models are adjusted for age, sex, and education

^c^Adjusted means for count outcomes are estimated using negative binomial regression

^d^ Adjusted means for continuous outcomes are estimated using generalized linear regression

^e^ Higher values of all outcomes indicates poorer health. All difference are calculated as Intervention mean – Control mean, negative scores indicate lower scores among the Intervention group.

**Supplementary Table 2.**

*Adjusted Means and Mean Differences in Primary Outcomes by Group Assignment & Sex, n=362*

|  | **Among Men, *n*=86** | | | **Among Women, *n*=275** | | |
| --- | --- | --- | --- | --- | --- | --- |
|  | **Intervention** | **Control** |  | **Intervention** | **Control** |  |
|  | ***n* = 46** | ***n* = 40** |  | ***n* = 115** | ***n* = 160** |  |
|  | **Adjusted^a^ Mean**  **(95% CI)** | **Adjusted^a^ Mean**  **(95% CI)** | **Difference^c-e^**  **(95% CI), *p*-value** | **Adjusted^a^ Mean**  **(95% CI)** | **Adjusted^a^ Mean**  **(95% CI)** | **Difference^c-e^**  **(95% CI), *p*-value** |
| **Healthcare Utilization** |  |  |  |  |  |  |
| Emergency Department Visits (count)^c^ | 1.40  (0.92, 2.11) | 1.53  (0.99, 2.36) | -0.089 (-0.633, 0.454), p = 0.7470 | 1.12  (0.84, 1.49) | 1.35  (1.08, 1.67) | -0.185 (-0.528, 0.158), p = 0.2914 |
| Hospital Admissions (count)^c^ | 2.42  (1.31, 4.47) | 1.53  (0.74, 3.17) | 0.459 (-0.394, 1.312), p = 0.2916 | 2.11  (1.25, 3.57) | 2.72  (1.76, 4.22) | -0.255 (-0.898, 0.388), p = 0.4368 |
| **PROMIS-29^d^** |  |  |  |  |  |  |
| Physical Functioning | 32.3  (30.1, 34.5) | 34.0  (31.7, 36.2) | -1.614 (-4.424, 1.196), p = 0.2564 | 33.7  (31.4, 35.0) | 34.6  (33.5, 35.6) | -0.878 (-2.459, 0.704), p= 0.2755 |
| Anxiety | 54.8  (51.6, 57.9) | 54.6  (51.3, 57.9) | 0.146 (-3.874, 4.166), p = 0.9427 | 54.3  (52.5, 52.8) | 54.3  (52.8, 55.8) | -0.024 (-2.237, 2.147), p = 0.9677 |
| Depression | 52.1  (49.0, 55.2) | 49.8  (46.6, 53.0) | 2.261 (-1.682, 6.205), p = 0.2571 | 51.0  (49.3, 52.8) | 52.1  (50.7, 53.5) | -1.059 (-3.169, 1.051), p = 0.3241 |
| Fatigue | 52.7  (49.8, 55.5) | 54.2  (51.2, 57.3) | -1.580 (-5.290, 2.130), p = 0.3992 | 51.2  (49.4, 53.0) | 54.5  (53.0, 56.0) | -3.288 (-5.457, -1.120), p = 0.0031 |
| Sleep | 52.1  (50.8, 53.4) | 53.1  (51.7, 54.4) | -0.954 (-2.652, 0.744), p = 0.2667 | 51.8  (51.2, 52.5) | 52.5  (52.0, 53.0) | -0.648 (-1.416, 0.120), p = 0.0976 |
| Social | 37.7  (35.1, 40.4) | 41.0  (38.2, 43.8) | -3.227 (-6.673, 0.219), p = 0.0661 | 40.2  (28.4, 42.0) | 40.7  (39.3, 42.2) | -0.486 (-2.664, 1.693), p = 0.6611 |
| Pain Interference | 56.4  (53.1, 59.6) | 58.6  (55.1, 62.0) | -2.206 (-6.479, 2.067), p = 0.3072 | 58.9  (56.8, 60.9) | 60.0  (58.3, 61.7) | -1.114 (-3.592, 1.363), p = 0.3766 |
| Pain Intensity | 4.7  (3.9, 5.5) | 5.3  (4.5, 6.2) | -0.624 (-1.700, 0.451), p = 0.2516 | 5.4  (4.9, 5.9) | 5.6  (5.2, 6.1) | -0.219 (-0.840, 0.401), p = 0.4867 |
| **Cardiac Symptom Experience^d^** | 1.56  (0.45, 2.68) | 3.01  (1.85, 4.18) | -1.450 (-2.881, -0.019), p=0.0472 | 2.18  (1.50, 2.88) | 2.79  (2.22, 3.35) | -0.596 (-1.435, 0.242), p = 0.1627 |

*n=1 is missing sex (control group)

^a^All models are adjusted for age, and education

^b^Adjusted odds ratios for ordinal outcomes are estimated using ordinal logistic regression

^c^Adjusted means for count outcomes are estimated using negative binomial regression

^d^Adjusted means for continuous outcomes are estimated using generalized linear regression

^e^Higher values of all outcomes indicates poorer health. All difference are calculated as Intervention mean – Control mean, negative scores indicate lower scores among the Intervention group.

**Supplementary Table 3.**

*Baseline Demographic and Health Characteristics by Group Assignment & and Study Completion Status, n=453*

| **Variable** | **Overall** | | | | | **Assigned to Intervention Group** | | | | | **Assigned to Control Group** | | | | |
| --- | --- | --- | --- | --- | --- | --- | --- | --- | --- | --- | --- | --- | --- | --- | --- |
|  | **Completers**  **(*n* = 362)** | | **Non-Completers**  **(*n* = 91)** | |  | **Completers**  **(*n* = 159)** | | **Non-Completers (*n* = 67)** | |  | **Completer s (*n* = 201)** | | **Non-Completers (n = 24)** | |  |
|  | ***n*** | **% or mean (SD)** | ***n*** | **% or mean (SD)** | ***p*-value** | ***n*** | **% or mean (SD)** | ***n*** | **% or mean (SD)** | ***p*-value** | ***n*** | **% or mean (SD)** | ***n*** | **% or mean (SD)** | ***p*-value** |
| Covariates (at Baseline) |  |  |  |  |  |  |  |  |  |  |  |  |  |  |  |
| Age in Years | 360 | 65.5 | 90 | 64.9 | 0.1978 | 159 | 66.2 | 67 | 63.8 | 0.0832 | 201 | 65.0 | 23 | 67.9 | 0.1199 |
| Sex |  |  |  |  |  |  |  |  |  |  |  |  |  |  |  |
| Male | 86 | 23.8 | 32 | 35.2 | 0.0277 | 46 | 28.6 | 27 | 40.3 | 0.0838 | 40 | 19.9 | 5 | 20.8 | 0.9233 |
| Female | 275 | 76.0 | 59 | 16.3 |  | 115 | 71.4 | 40 | 59.7 |  | 160 | 79.6 | 19 | 79.2 |  |
| Race |  |  |  |  |  |  |  |  |  |  |  |  |  |  |  |
| Hispanic | 2 | 0.6 | 2 | 2.2 | 0.0817 | 1 | 0.6 | 1 | 1.5 | 0.6116 | 1 | 0.5 | 1 | 4.2 | 0.0626 |
| Non-Hispanic Black | 298 | 82.3 | 82 | 90.1 |  | 134 | 83.2 | 59 | 88.1 |  | 164 | 81.6 | 23 | 95.8 |  |
| Non-Hispanic White | 18 | 5.0 | 2 | 2.2 |  | 8 | 5.0 | 2 | 3.0 |  | 10 | 5.0 | 0 | 0.0 |  |
| Other | 37 | 10.2 | 4 | 4.4 |  | 16 | 9.9 | 4 | 6.0 |  | 21 | 10.4 | 0 | 0.0 |  |
| Education |  |  |  |  |  |  |  |  |  |  |  |  |  |  |  |
| < High School | 36 | 9.9 | 17 | 18.7 | 0.0018 | 12 | 7.5 | 11 | 16.4 | 0.0422 | 24 | 11.9 | 6 | 25.0 | 0.0015 |
| High School Grad or GED | 82 | 22.7 | 29 | 31.9 |  | 38 | 23.6 | 17 | 25.4 |  | 44 | 21.9 | 12 | 50.0 |  |
| Some College | 157 | 43.4 | 37 | 40.7 |  | 74 | 46.0 | 33 | 49.3 |  | 83 | 41.3 | 4 | 16.7 |  |
| College Graduate or higher | 85 | 23.5 | 8 | 8.8 |  | 35 | 21.7 | 6 | 9.0 |  | 50 | 24.9 | 2 | 8.3 |  |
| Self-rated health |  |  |  |  |  |  |  |  |  |  |  |  |  |  |  |
| Excellent | 7 | 1.9 | 1 | 1.1 | 0.8448 | 1 | 0.6 | 1 | 1.5 | 0.8857 | 6 | 3.0 | 0 | 0.0 | 0.103 |
| Very Good | 33 | 9.1 | 8 | 8.8 |  | 14 | 8.7 | 8 | 11.9 |  | 19 | 9.5 | 0 | 0.0 |  |
| Good | 118 | 32.6 | 28 | 30.8 |  | 57 | 35.4 | 22 | 32.8 |  | 61 | 30.3 | 6 | 25.0 |  |
| Fair | 137 | 37.8 | 40 | 44.0 |  | 64 | 39.8 | 25 | 37.3 |  | 73 | 36.3 | 15 | 62.5 |  |
| Poor | 66 | 18.2 | 14 | 15.4 |  | 24 | 14.9 | 11 | 16.4 |  | 42 | 20.9 | 3 | 12.5 |  |
| Heart disease dx (can select more than 1) |  |  |  |  |  |  |  |  |  |  |  |  |  |  |  |
| Atrial Fibrillation | 73 | 20.2 | 13 | 14.3 | 0.2011 | 32 | 19.9 | 10 | 14.9 | 0.3797 | 41 | 20.4 | 3 | 12.5 | 0.3565 |
| Myocardial Infarction | 56 | 15.5 | 23 | 25.3 | 0.0276 | 26 | 16.1 | 15 | 22.4 | 0.2634 | 30 | 14.9 | 8 | 33.3 | 0.0229 |
| Valvular Disease | 31 | 8.6 | 3 | 3.3 | 0.0883 | 9 | 5.6 | 3 | 4.5 | 0.7318 | 22 | 10.9 | 0 | 0.0 | 0.0879 |
| Pulmonary Hypertension | 11 | 3.0 | 7 | 7.7 | 0.0422 | 6 | 3.7 | 5 | 7.5 | 0.2304 | 5 | 2.5 | 2 | 8.3 | 0.1190 |
| Angina | 59 | 16.3 | 18 | 19.8 | 0.4292 | 21 | 13.0 | 12 | 17.9 | 0.3414 | 38 | 18.9 | 6 | 25.0 | 0.4768 |
| Congestive Heart Failure | 66 | 18.2 | 12 | 13.2 | 0.2545 | 24 | 14.9 | 9 | 13.4 | 0.7732 | 42 | 20.9 | 3 | 12.5 | 0.3311 |
| Peripheral Vascular Disease | 45 | 12.4 | 8 | 8.8 | 0.3342 | 21 | 13.0 | 6 | 9.0 | 0.3841 | 24 | 11.9 | 2 | 8.3 | 0.6014 |
| Other Heart Disease Condition | 12 | 3.3 | 3 | 3.3 | 0.9931 | 7 | 4.3 | 2 | 3.0 | 0.6302 | 5 | 2.5 | 1 | 4.2 | 0.6294 |
| Any Heart Disease | 199 | 55.0 | 48 | 52.7 | 0.7032 | 84 | 52.2 | 35 | 52.2 | 0.9929 | 115 | 57.2 | 13 | 54.2 | 0.7757 |
| Heart disease dx (can select more than 1), Sum | 360 | 1.0 | 90 | 1.0 | 0.8043 | 161 | 0.91 | 67 | 0.93 | 0.9114 | 201 | 1.03 | 24 | 1.04 | 0.9640 |
| Risk factors (can select more than 1) |  |  |  |  |  |  |  |  |  |  |  |  |  |  |  |
| High Cholesterol | 281 | 77.6 | 73 | 80.2 | 0.5923 | 130 | 80.7 | 54 | 80.6 | 0.9794 | 151 | 75.1 | 19 | 79.2 | 0.6632 |
| High Blood Pressure | 335 | 92.5 | 79 | 86.8 | 0.0816 | 154 | 95.7 | 56 | 83.6 | 0.0021 | 181 | 90.0 | 23 | 95.8 | 0.3573 |
| Diabetes | 162 | 44.8 | 35 | 38.5 | 0.2793 | 83 | 51.6 | 26 | 38.8 | 0.0792 | 79 | 39.3 | 9 | 37.5 | 0.8641 |
| Chronic Kidney Disease | 21 | 5.8 | 5 | 5.5 | 0.9105 | 6 | 3.7 | 4 | 6.0 | 0.4511 | 15 | 7.5 | 1 | 4.2 | 0.5526 |
| Currently Smoke | 96 | 26.5 | 37 | 40.7 | 0.0113 | 33 | 20.5 | 30 | 44.8 | 0.0002 | 63 | 31.3 | 7 | 29.2 | 0.7676 |
| Any Heart Disease Risk Factor | 320 | 88.4 | 81 | 89.0 | 0.7495 | 153 | 95.0 | 59 | 88.1 | 0.0200 | 167 | 83.1 | 22 | 91.7 | 0.3904 |
| Risk factors (can select more than 1), Sum | 362 | 2.5 | 91 | 2.5 | 0.5251 | 159 | 2.52 | 67 | 2.54 | 0.9050 | 196 | 2.44 | 24 | 2.15 | 0.9217 |
| Comorbidities |  |  |  |  |  |  |  |  |  |  |  |  |  |  |  |
| Depression | 137 | 37.8 | 34 | 37.4 | 0.9031 | 65 | 40.4 | 26 | 38.8 | 0.7987 | 72 | 35.8 | 8 | 33.3 | 0.7967 |
| Cancer | 57 | 15.7 | 13 | 14.3 | 0.7451 | 26 | 16.1 | 9 | 13.4 | 0.6214 | 31 | 15.4 | 4 | 16.7 | 0.8818 |
| Prediabetes | 155 | 42.8 | 33 | 36.3 | 0.2247 | 80 | 49.7 | 24 | 35.8 | 0.0505 | 75 | 37.3 | 9 | 37.5 | 0.9712 |
| Stroke | 61 | 16.9 | 14 | 15.4 | 0.7133 | 24 | 14.9 | 9 | 13.4 | 0.7467 | 37 | 18.4 | 5 | 20.8 | 0.7820 |
| Arthritis | 244 | 67.4 | 60 | 65.9 | 0.8136 | 105 | 65.2 | 41 | 61.2 | 0.5642 | 139 | 69.2 | 19 | 79.2 | 0.2122 |
| Chronic obstructive pulmonary disease | 91 | 25.1 | 30 | 33.0 | 0.1385 | 31 | 19.3 | 19 | 28.4 | 0.1331 | 60 | 29.9 | 11 | 45.8 | 0.1234 |
| Asthma | 98 | 27.1 | 23 | 25.3 | 0.7601 | 42 | 26.1 | 15 | 22.4 | 0.6199 | 56 | 27.9 | 8 | 33.3 | 0.6168 |
| Sum of comorbidities | 362 | 2.3 | 91 | 2.27 | 0.2342 | 161 | 2.32 | 67 | 2.13 | 0.3609 | 201 | 2.3 | 24 | 2.7 | 0.2879 |
| Intervention Dosage | 161 | 5.8 | 45 | 1.4 | <0.0001 | 161 | 5.74 | 45 | 1.2 | <0.0001 | --- |  | --- | --- | NA |

Note. GED = General educational development.
